# Ultrasound assessment of the median nerve: a biomarker that can help in setting a treat to target approach tailored for carpal tunnel syndrome patients

**DOI:** 10.1186/s40064-014-0779-4

**Published:** 2015-01-13

**Authors:** Yasser El Miedany, Maha El Gaafary, Sally Youssef, Ihab Ahmed, Annie Nasr

**Affiliations:** Rheumatology, Darent Valley Hospital, Dartford, Kent UK; Rheumatology and Rehab, Department, Ain Shams University, Cairo, Egypt; Department of Community, Environmental and Occupational Medicine, Ain Shams University, Cairo, Egypt; Internal Medicine, Cairo University, Cairo, Egypt; Radiology Department, Ain Shams University, Cairo, Egypt

**Keywords:** US, Carpal tunnel syndrome, Median nerve, Tenosynovitis

## Abstract

Ultrasonography (US) is a valuable tool for confirming the diagnosis of carpal tunnel syndrome (CTS) as it enables the detection of changes in the median nerve shape and rule out anatomic variants as well as space-occupying lesions such as ganglion cysts or tenosynovitis. This work was carried out aiming at: 1. Ultrasonography assessment of the median nerve and its neurovascular blood-flow in CTS patients before and after management. 2. Verify the possibility of using baseline US parameters as a biomarker to predict likely outcomes and frame a treatment plan for CTS patients.

233 CTS subjects diagnosed based on clinical and electrophysiological (NCS) testing were included in this work. US measures at the tunnel inlet included: cross sectional area, flattening ratio and neural Power Doppler (PD) signals. Patients who had severe NCS outcomes or neurological deficit were referred for open surgical decompression; the remaining patients were given the choice of either conservative or surgical management. The main outcome variable was improvement >70% in CTS symptoms. Assessments were carried out at baseline, 1-week, 1-month and 6-months post treatment. Results revealed an inverse relation between the neural vasculature and CTS severity defined by NCS (r = − 0.648). In CTS cases treated conservatively, the US measures started to improve within 1-week, whereas in the surgically treated cohort there was an initial phase of post-operative nerve measures increase, before settling at 1-month time of follow-up. The risk of poor outcomes was significantly higher (RR 3.3) in patients with high median nerve flattening ratio. This risk was most marked in the cohort with nerve flattening associated with longer duration of illness (RR 4.3) and low PD signal (RR 4.1). The results revealed that in addition to the diagnostic value of US in CTS, the detection of increased median nerve neuro-vasculature has a good prognostic value as an indicator of early median nerve affection.

## Introduction

Carpal tunnel syndrome (CTS), first reported by Phalen (1966), is one of the most common peripheral mono-neuropathies which occurs due to localized compression of the median nerve (MN) in the CT. With the introduction of new technology including computers, mobile phones and tablets, its prevalence increased significantly and it has been reported to represent up to 90% of all entrapment neuropathies (El Miedany et al. 2004; Aroori and Spence 2008; Mohammadi et al. 2010). Evaluation of patients presenting with CTS manifestations has long relied on their clinical symptoms as well as nerve conduction studies (NCS). Although standard symptoms and positive provocative testing may enable identifying acute cases, the subjectivity and sensitivity of these measures results in very poor reliability and diagnostic accuracy (Kuhlman and Hennessey 1997; Salerno et al. 2000; Mondelli et al. 2001; El Miedany et al. 2008). Similarly, though studies revealed sensitivity and specificity data in favour of electrodiagnostic testing for the CTS diagnosis (Jordan et al. 2002; El Miedany et al. 2007; Strickland and Gozani 2011), abnormal NCS results do not necessarily equate to the correct diagnosis. Earlier reports showed that NCS can be normal in early cases (El Miedany et al. 2007). Furthermore, NCS were reported as not sensitive to change or management, hence, a poor predictor of treatment outcomes (Evans et al. 2012).

Inspite of some limitations, ultrasonography (US) was found to be a good tool not only for the CTS diagnosis, but also for identifying the MN affection severity. In comparison to NCS, US measurements had a sensitivity of 29.4% to 100% and specificity of 47% to 100% (Roll et al. 2011). On the other hand, MN hypervascularity showed sensitivity and specificity of 50% (Wang et al. 2008) and 65% (Mallouhi et al. 2006) respectively. Better accuracy was suggested by the combination of sonographic measures of the MN cross-sectional area (CSA), and hypervascularity, which resulted in 90% concordance with the CTS clinical presentation (Rahmani et al. 2011).

The search for markers identifying key targets for the assessment of major outcomes in musculoskeletal diseases has become one of the hot issues in rheumatology. Possible markers should be objectively measured, indicatory of normal biology as well as the pathologic process, indicator of response to therapy and prognosis. It should also be a good indicator of modification of the pathological process and help to identify (in early cases) the patients who are going to respond quickly to therapy with the vision to tailor management to the patient status (Ferraccioli et al. 2013). So far this target has not been achieved in CTS. There have been many studies investigating US changes following CTS management, however, few ones were conducted to follow-up the changes (both NCS and US) (Jeong et al. 2011). The novel concept of using US assessment as a biomarker which can help in setting a “Treat to Target” approach tailored for CTS patients, expands the scope of using this tool in clinical practice. In this report we present a large prospective US study of CTS patients. The specific aim of the study was to assess the median nerve both by Gray-scale US and neural vascular flow (using Power Doppler (PD) before and after management. Moreover, we investigated the feasibility of initial US parameters for setting up a treatment plan tailored to the patient’s needs and its ability to predict treatment outcomes.

## Results

### Results of patient baseline assessment

233 subjects (81% of those eligible for this study) completed the 6-months follow-up period. The mean age of patients enrolled in the cohort was 53.7 + 3.71 years and 60% of the patients cohort (140/233 patients) were female. The mean (SD) duration of clinical symptoms was 10 + 2.14 months (min 3 months and maximum 19 months) months. There was no significant difference on comparing age and sex distribution in both CTS patient cohort and the control group. Analysis of CTS duration of Illness patients stratified according to grade of CTS, revealed significantly (p < .001) longer disease duration in the severe subgroup (Median (IQR) was 15 (7.0) months), in comparison to 7 (6.0) months in the mild group and 8 (15.0) months in the moderate subgroup.

### Patient reported management outcomes (PROMs)

115/233 (49.4%) of the patient cohort were treated conservatively (i.e. local injection, splints and exercise program, whereas 118/233 (50.6%) were treated surgically. 81% (93/115) of the patients, treated conservatively improved significantly within 1–4 weeks and maintained their improvement throughout the 6-months follow-up period. On the other hand, 78% (92/118) of the patients treated surgically took between 1–6 months to show similar improvement. Overall satisfaction (>70% improvement on the global scale was reported in 51/95 (53.7%) in the mild group versus 22/76 (28.9%) in the moderate group and 11/60 (18.3%) in the severe group at week-1 of follow up (p < 0.001). This improved, at 6-month post management, to 87/118 (73.7%) in the mild group, 53/76 (69.7%) in the moderate group and 38/60 (63.3%) in the severe group. Symptom severity scores improved from a pre-management average of 7.3 (SD 0.61) to 2.25 + 0.42 at 6-months, whereas functional limitation scores improved from 3.8 + 0.37 to 1.2 + 0.26. These differences between pre-management and follow-up symptom severity and functional limitation scores were highly statistically significant (P <0.001 for both).

### Nerve conduction outcomes in response to management

Stratifying patients according to NCS outcomes revealed severe MN compression (grade 5 and 6) in 60/233 (25.8%) patients, moderate (grade 3 and 4) in 76/233 (32.6%), and mild (grade 1 and 2) in 118/233 (50.6%). At baseline, there was significant difference (p < 0.001) on comparing the distal sensory and motor latencies as well as the nerve conduction velocity in the 3 subgroups. Also there were significant difference between the patients and the control groups regarding these 3 variables. There was a significant correlation between the main NCS outcomes and US measures (Table 1). On comparing pre- and post- management NCS, there was significant difference in the mild CTS group (P < 0.01); whereas in the moderate group, though the post-management NCS outcomes did not meet the normal ranges, the improvement of the NCS figures remained significant (P < 0.05). In contrast, the improvement in the NCS outcomes did not achieve significant difference the severe group.

**Table 1:** **Correlation between Nerve conduction study parameters and US outcome measures at baseline**

	**Distal Sensory Latency**	**Distal Motor Latency**	**Nerve Conduction Velocity**
Cross Sectional Area	0.854*	0.759*	−0.867*
Flattening Ratio	0.533*	0.734*	−0.811*
Power Doppler (PD) Score	0.625*	0.349*	−0.334*

*: P< 0.01.

### US outcomes

CSA, FR and PD score (>2) were the 3 main US outcome measures which showed a significant relation both at baseline and after treatment. There was significant inverse relation (r = −0.372, P < 0.03) between PD and disease duration. Enhanced vascularity (PD score >2) was seen in 66/97 (68%) of the patients with disease duration < 6 months, whereas it was seen in 2/59 (3.4%) of the patients with disease duration > 15- months. There was also an inverse relationship between intra-neural vascular flow and CTS severity based on NCS (r = −0.737) as well as the presence of neurological manifestations (r = −0.642). The MN sonographic changes in CTS can be divided into 3 phases: Phase 1 “Hypervascularity”: PD score of 2 or greater. Initially, there was enhanced peri-neural vascularity which extended later to intra-neural hypervascularity. Hypervascularity was seen in 26/50 (52%) of the patients who had mild (grade 1 and 2) CTS, whereas it was present in 2/59 (3.4%) in severe CTS (grade 5 and 6) diagnosed according to NCS. Phase 2 “Nerve edema”: swelling of the MN at the CT entrance manifested by CSA > 10 mm2. The median nerve CSA was significantly correlated with duration of symptoms (r = 0.726. P < 0.01). In patient with disease duration < 6 months, the mean CSA was 11.3 + 0.3 mm2, in patients with disease duration between 6–12 months mean CSA 14.04 + 0.4 mm2, whereas in patients with disease duration > 12 months, CSA mean was 18.1 + 0.3 mm2. Phase 3 “nerve flattening”: Flattening was significantly correlated (r = 0.516, p < 0.01) to severity of the MN compression, identified by NCS. In mild MN compression, the mean FR was 2.5, and in moderate nerve compression the ratio was 2.8, whereas in severe compression it was 2.91. Table 2 shows the mean of the 3 US outcome measures in the CTS patients cohort stratified according to the severity of their NCS before and after treatment.

**Table 2:** **Mean and standard deviation of the 3 US outcome measures in the CTS patients cohort stratified according to the severity of their NCS before and after treatment**

**Median Nerve**	**Nerve Conduction Testing**	**Baseline**	**1-wk post- management**	**1-month post management**	**6-month post- management**	**P-value**
Cross Sectional Area (CSA)	Mild	11.34 (0.16)	10.92 (0.2)	9.81 (0.15)	9.78 (0.3)	<0.001*
	Moderate	14.4 (0.5)	15.4 (0.4)	11.1 (0.3)	11.09 (0.4)	<0.001*
	Severe	18.91 (0.7)	18.87 (0.6)	17.43 (0.7)	16.41 (0.5)	<0.001*
Flattening Ratio	Mild	2.52 (0.1)	2.33 (0.3)	2.2 (0.4)	2.02 (0.3)	<0.001*
	Moderate	2.84 (0.02)	2.6 (0.06)	2.47 (0.04)	2.42 (0.03)	<0.001*
	Severe	2.9 (0.2)	2.89 (0.4)	2.88 (0.3)	2.78 (0.2)	<0.001*
Power Doppler	Mild	1.28 (0.6)	0.2 (0.4)	0.04 (0.2)	0.09 (0.3)	<0.001*
	Moderate	2.1 (0.5)	1.08 (0.6)	0.49 (0.5)	0.05 (0.2)	<0.001*
	Severe	0.40 (0.5)	0.4 (0.5)	0.33 (0.5)	0.30 (0.46)	<0.001*

*: P< 0.01.

### US outcomes in response to management

PD was the first US outcome measure to show significant change in response to management as early as 1-week. This improvement remained till 1-month post-treatment, the process then slowed down before platauing. Table 3 shows that the CSA and flattening ratio improved significantly (back to normal range in patients with mild or mild-moderate nerve compression, whereas they remained unchanged in the severely compressed cases. In contrast with NCS which did not show any significant correlation with the treatment response at 6-months, there was significant correlation (p < 0.01) between baseline US variables, namely CSA, Flattening ratio and PD, and the percentage of improvement at 1st week, 1st month and 6-months post-treatment.

**Table 3:** **Correlation between “Baseline US findings” and “% improvement of the US parameters” measured at 1st week, 1st month and 6th month**

**US measure**	**1st wk**	**1st mth**	**6th mth**
Cross Sectional Area	−0.649*	−0.195*	−0.253*
Flattening Ratio	−0.635*	−0.144*	−0.233*
Flexor Retinaculum	−0.062	−0.068	−0.015
Power Doppler (PD) Score	0.632*	0.499*	0.264*

*: P< 0.01.

### Multivariate model

**Figure 1:**
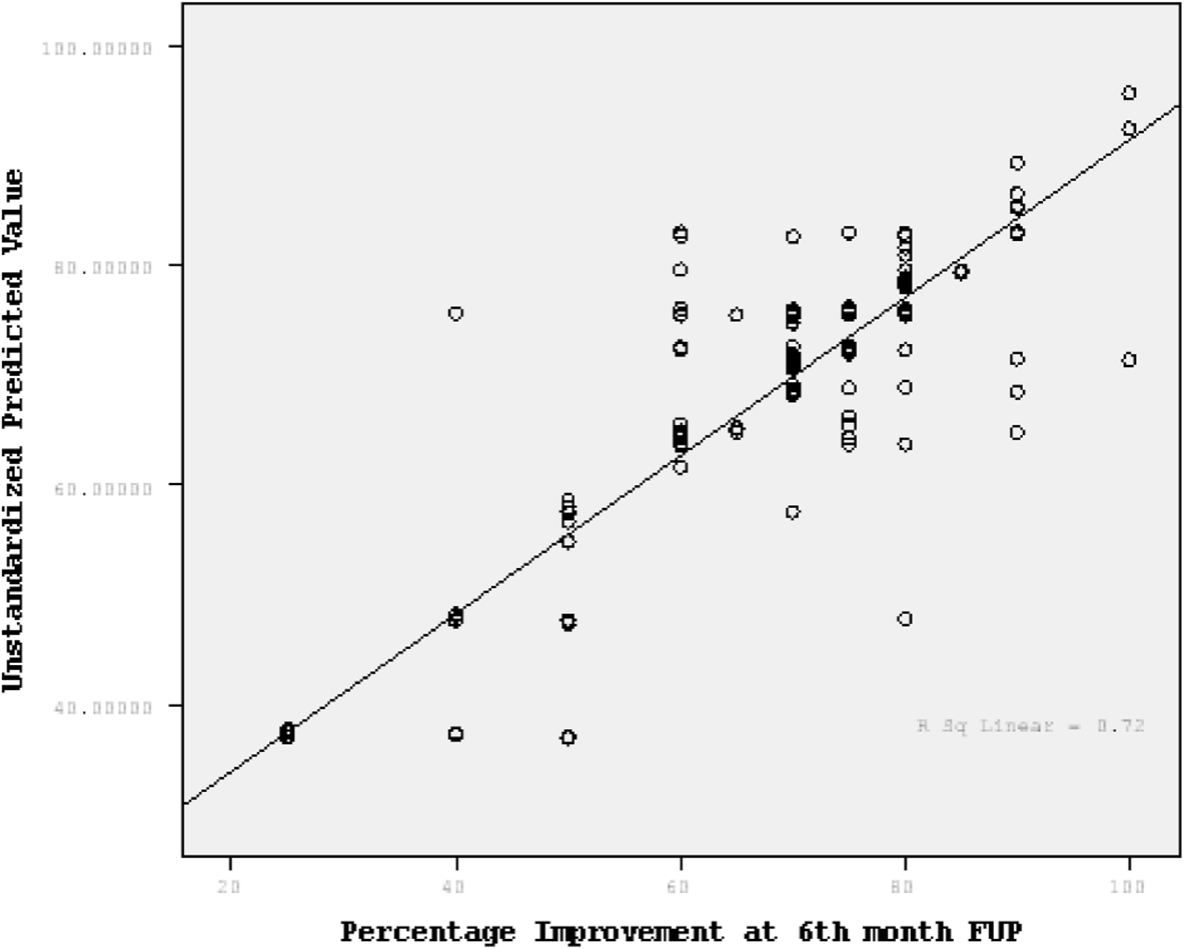
**Scatterplot displaying Actual Percent Improvement at 6 months FUP and Unstandardized Predicted Values of the Model.**

**Figure 2:**
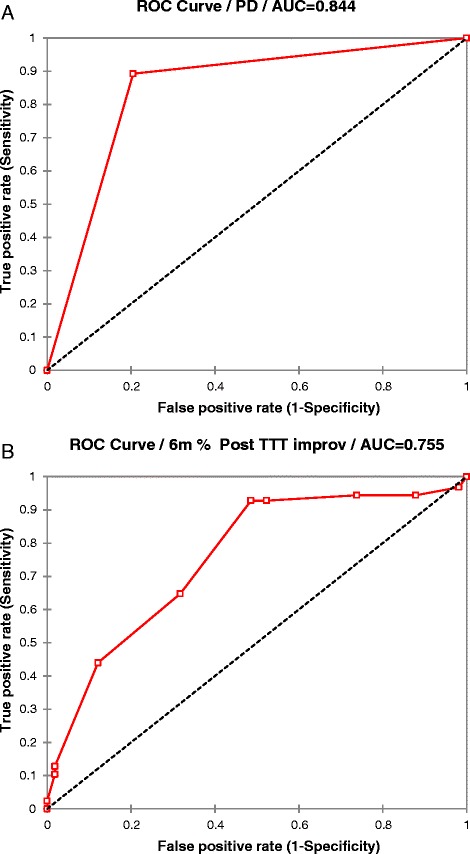
**ROC of using Power Doppler (PD) as a predictor of good outcome at 6-months of follow up in patients with disease duration less than 6 months (a) and in the assessed patients independent of their disease duration (b).**

In multivariate regression analyses involving the entire cohort, greater pre-management CSA (>14.0 mm2) and Flattening ratio (>2.8) were the most important predictors of poorer outcome and presence of symptoms at 6-months follow-up. Table 4 displays predictors of percent improvement at 6 months follow up. Baseline CSA (<14.0 mm2) and PD score (>2) at baseline were associated with better response at 6 months. Figure 1 is a scatterplot displaying actual percent improvement at 6 months follow-up. Figure 2 is an ROC assessing the potential use of PD as a predictor of good outcome (>70% improvement of the patient global score). Independent of the disease duration, AUC was 0.755 with sensitivity of 92.8% and specificity of 51.4% and positive likelihood ratio of 1.91, whereas in the patient cohort with disease duration < 6 months, AUC was 0.844 with sensitivity of 89.3% and specificity 79.5% and positive likelihood ratio of 4.353.

**Table 4:** **Multivariate linear regression analysis displaying predictors of percent improvement at 6 months follow up**

**Variables in the equation**	**Unstandardized B (SE)**	**P value**
Constant	39.004 (6.2)	<0.001*
Baseline Cross Sectional Area (CSA)	−6.67 (2.06)	0.001*
Baseline PD (score >2)	0.699 (0.03)	<0.001*
R^2^	0.717	

*: P< 0.01.

### Reproducibility of ultrasound findings

The ICC of CSA (0.90; 95% CI 0.79 to 0.95) and flattening ratio (0.85; 95% CI 0.8 to 0.9), were high. The linear weighted kappa coefficient for power Doppler assessments was 0.78 (95% CI 0.76 to 0.81). The ICC for intra-observer variability was 0.85 (95% CI 0.84 to 0.91).

## Discussion

This study was carried out to assess the possibility of using musculoskeletal US assessment of the median nerve as a biomarker and whether it can be of help to set a “Treat to Target” management plan tailored the patient’s status. This work supports the concept of US being a rapid, safe, and inexpensive technique which can be useful to assess CTS patients both at baseline as well as to assess their response to treatment. In comparison to the control group, the 3 US outcome measures, PD, CSA and nerve flattening ratio have shown high diagnostic accuracy and reliability which make them suitable for the diagnosis of CTS, but also as a staging tool and predictor of treatment outcome. There was an inverse relation between the median nerve neural vasculature (PD score >2) and disease duration, presence of neurological deficit as well as CTS severity (based on nerve conduction results). The 3 US outcome measures also correlated significantly with the nerve conduction study measures (namely, distal sensory latency, distal motor latency and nerve conduction velocity). Whilst, PD (score >2) and cross sectional area < 14 mm^2^ were predictors of good outcome in response to management, the risk of a poor outcome was significantly higher in the patients with high median nerve flattening ratio at the carpal tunnel inlet, cross sectional area >14 mm^2^ and low PD signal (score <2).

The significant inverse relation between PD signal and disease duration of the disease and the presence of neurological deficits, reflect the state of vasculature stasis early during the disease process. The increased venous vasculature starts at the peri-neural sheath first then extends to involve also the intra-neurium and consequently lead to increase in the cross sectional area as a result of the nerve edema. These findings are in agreement with earlier published data (Lundborg et al. 1983; Mackinnon et al. 1984; Lundborg and Dahlin 1996) suggesting that the persistent edema and increased interstitial pressure eventually lead to increase axonal transport and intra-neural blood flow followed by fibroblastic activity and scar formation in and about the nerve. This would explain the flattening state of the median nerve reported in this study in the more severe cases and the patients with long disease duration.

Whilst the use of US to monitor response to therapy focussed mainly on surgical decompression, the interpretation of the reported US outcome measures have been contradictory. In most of the earlier published research (Lee et al. 2005; Hammer et al. 2006; Abicalaf et al. 2007; Colak et al. 2007; Smidt and Visser 2008), a significant decrease was recorded in the cross-sectional area of the median nerve at the tunnel inlet ranging between 1 and 3 mm^2^. In the study carried out by Mondelli et al. (2008b), a correlation was reported between the degree of reduction of the cross-sectional area after surgery and both improvement of symptoms as well as results of nerve conduction studies. On the other hand, in the study carried out by Naranjo et al. (2010), no association between clinical improvement and the reduction of the median nerve cross-sectional area after surgery was found. Stratifying the patients based on the severity of the median nerve compression, carried out in this work, has helped to clarify this contradiction. Earlier in the disease process and in patients with mild or mild-moderate median nerve compression, there was significant reduction of the median nerve cross sectional area as well as the PD score. This improvement in the US outcome measures was significantly associated with the improvement of the patients’ symptoms. However, later in the disease course, and in severe cases, where cross sectional area exceeded 14 mm^2^ and the nerve got significantly flattened, whilst there was some improvement in the cross sectional area, this was not associated with improvement of the nerve morphology or the patients’ symptoms. This finding highlights the importance of early intervention for CTS patients before significant nerve damage occurs.

The published literature on predictors of outcomes of surgery has some methodological limitations. Most of the research studies implemented retrospective paradigms, impeding expressive examination of pre-operative important aspects such as the severity of the patient-reported symptoms as well as functional disability. On the other hand, few studies implemented standardized outcome measures which matter most to patients such as symptoms relief, improvement of the functional disability, as well as satisfaction (Levine et al. 1993; Bessette et al. 1997; El Miedany et al. 2014). In general, a narrow array of potential predictors, focused mainly on the prognostic significance of physiologic impaired parameters such as aberrations on neurophysiological assessment and physical examination, have been traditionally considered by most of the research studies. This work was set up based on the analysis of the baseline parameters in association with the clinical as well as patient-reported management outcomes. This enabled overcoming several of the aforementioned limitations. A multidimensional model of predictors was implemented including patient-reported management outcomes, severity of symptoms, functional disability as well as patients’ satisfaction. Results of this work showed that the patient-reported outcome measures, which represent the key indicators of successful management approach from the patient’s point of view Katz et al. 1994; Bessette et al. 1997), were sensitive to change and correlated significantly to changes in the US outcome measures starting from the first week after treatment. This concurs with the earlier findings reported by Katz et al. (2001), that patient reported outcome measures were not only a valid management outcome measure, but also a robust prognosticator of less favourable management outcome.

An accurate assessment of predictors of outcomes for carpal tunnel syndrome management would enable the treating physicians as well as patients to make more informed decisions about treatment approach whether conservative or surgical, and to develop more precise anticipation of outcomes. Several earlier studies have explored the factors influencing the treatment outcomes of carpal tunnel syndrome, in particular surgical decompression. Poor management outcomes have been associated with pre-operative neurological deficit such as muscle weakness or atrophy (Muhlau et al. 1984), worker’s reimbursement (Higgs et al. 1995), exposure to vibration (Hagberg et al. 1991), repetitive or heavy manual work (Yu et al. 1992), predisposing medical conditions (Kulick et al. 1986) including thyroid disease and diabetes mellitus (De Stefano et al. 1997), improper diagnosis (Eason et al. 1985), and incomplete sectioning of the flexor retinaculum (Eason et al. 1985). Symptoms duration has been a matter of controversy, as in some studies symptoms duration were not associated with the management outcomes (Al-Qattan et al. 1994), whereas in others, worse outcomes were reported in patients with longer symptoms duration (De Stefano et al. 1997). Furthermore, the severity of the nerve conduction testing outcome has not been associated with the treatment outcomes (Yu et al. 1992; Al-Qattan et al. 1994). In contrast with US, nerve conduction studies identify only permanent nerve damage, but not intermittent nerve disturbance particularly in early CTS cases. Results of this work revealed that US helps in the early diagnosis of the condition, identifying the underlying pathological cause, the progress of the condition and its severity as well as response to management, which would make it fit with the definition a biomarker. These findings have important implications for clinical practice and future research. Clinicians should incorporate these results in their discussions with patients regarding the likely outcomes of carpal tunnel management. Patients should understand that having high PD score and mild to moderate increase in the CSA (<14 mm^2^) all signify a more favourable outcome to conservative management approach. This does not imply that patients with these risk factors should be denied surgery; rather, the patient and clinician should acknowledge that for complex reasons, outcomes are typically better in the presence of these factors. On the other hand, surgical decompression should be offered to the patients with significant flattening of the median nerve (>2.8) as well as cross sectional area (>14 mm^2^).

The methodological strengths of this study include the high rate of participation, acceptable rate of subject retention at follow-up, use of reliable and valid measures including patient reported outcome measures as well as nerve conduction testing, and the availability of potential predictors across numerous domains. Limitations that should be acknowledged include a relatively constrained set of potential workplace psychosocial predictors, perceived job strain, as well as availability of support at work.

## Conclusion

In addition to the diagnostic value of ultrasonography in CTS, US is a tool fit to be a radiologic biomarker for carpal tunnel syndrome. The detection of increased neural vasculature of the median nerve is an indicator of early median nerve affection and has a good prognostic value and is in favour of a conservative management approach, whereas flattened median nerve with significantly increase cross sectional area, warrant surgical decompression as a treatment option.

## Methods

### Study

Prospective case–control study which included consecutive patients attending the outpatient clinic and diagnosed to have CTS after clinical and neurophysiological assessment. Local ethical and methodological protocols’ approval for this study conduction were obtained. All the subjects who shared in the study signed an informed consent in keeping with the Declaration of Helsinki.

### Patients

Patients presenting with one or more of the following symptoms in at least one wrist were included: (1) paresthesias, pain and/or sensory deficits in the hand in a median nerve distribution; (2) nocturnal/ early morning worsening of paresthesias with disturbed sleep; (3) paresthesias relieved by hand movement or shaking; (4) pain and/or paresthesias in a median nerve distribution provoked by repetitive exercises; (5) weakness of fingers supplied by the median nerve.

Patients with previously diagnosed CTS, conditions resulting in an increased risk of (associated) CTS such as previous surgery at wrist, recent wrist fracture or traumatic nerve injury, known inflammatory rheumatic disease, metabolic disorders or pregnancy, patients with known polyneuropathy or contraindications to electrophysiological testing were excluded.

### Clinical assessment

Clinical examination consisted of an evaluation of muscular strength and trophic changes, sensory function and clinical provocation tests including Phalen’s, reverse Phalen’s and carpal tunnel compression test.

### Neurophysiological assessment

Nerve conduction studies (NCS) were performed at the symptomatic side(s) according to a routine protocol. Skin temperature over the dorsum of the hand was kept at 34°C. The sensory median nerve conduction velocity (normal values 50 m/s), distal sensory latency (baseline to peak amplitude from the second finger, with retrograde stimulation being conducted at the wrist (14 cm proximal area from the recording site) and the palm (7 cm proximal area from the recording site), distal motor latency (baseline to initial latency from the abductor pollicis brevis muscle center with stimulation performed at the wrist, 7 cm from the recording site) and median motor compound muscle action potential (5 mV) were determined (El Miedany et al. 2004).

The patients with abnormal NCS were classified according to electrodiagnostic grading (Bland 2000) into:

- **Grade 0:** Normal standard and comparative tests.
- **Grade 1:** Very mild CTS: Normal standard tests, abnormal comparative tests or reduced nerve conduction velocity of the median nerve across the carpal tunnel.
- **Grade 2:** Mild CTS: Abnormal sensory with a normal motor response that is, prolonged antidromic distal sensory latency (DSL) >3.6 ms to the second digit.
- **Grade 3:** Moderate CTS: Abnormal median sensory and motor response that is, prolonged distal motor latency to abductor pollicis brevis (APB) is >4.2 ms but <6.5 ms, and prolonged antidromic distal sensory latency with decreased amplitude sensory nerve action potential.
- **Grade 4:** Severe CTS: Absence of sensory response, abnormal distal motor latency to APB but still <6.5 ms with decreased amplitude of compound muscle action potential and abnormal EMG activity in abductor policis brevis muscle.
- **Grade 5:** Very severe CTS: Terminal latency to APB >6.5 ms.
- **Grade 6:** Extremely severe CTS: Absence of median motor and sensory responses (surface motor potential from APB <0.2 mV amplitude).

The final diagnosis was established based on symptoms, clinical evaluation and NCS results.

### US assessment

Ultrasonography studies were performed by two sonographers experienced in musculoskeletal sonography using a Mylab 25, Esaote, Italy US machine, with a multifrequence linear transducer 14–18 MHz. Both sonographers were unaware of clinical and NCS results. Patients were examined in the sitting position with hands resting in a horizontal supine position on the examination table with fingers semi-extended (Mondelli et al. 2008a).

A perpendicular angle of the probe was maintained during analysis to prevent anisotropy and median nerve deformation. imaging parameters were adjusted to maximise the contrast between examined structures. Power Doppler settings were standardised accordingly: frequency 11.9 MHz, pulse repetition frequency 600 Hz (lowest possible avoiding motion artefacts most of the time) and medium persistence.

The power Doppler gain was optimised by increasing gain until noise appeared and then reduced just enough to suppress the noise (Torp-Pedersen and Terslev 2008). The transducer was applied on the wrist with the minimal possible pressure to avoid any impact on the intra-neural vessels.

The Cross-sectional area (CSA) of the median nerve was measured by continuous tracing a continuous line at the inner hyper-echoic rim of the median nerve at the carpal tunnel inlet (at the level of the pisiform and scaphoid bones) (Ziswiler et al. 2005; Klauser et al. 2009). Images were magnified in order to reduce measurement error. The CSA of the median nerve measured was recorded. Flattening ratio defined as the ratio of the major axis of the median nerve to its minor axis was calculated.

Power Doppler signals were graded from 0 to 3, in which 0 represented no power Doppler signal, 1 = one single vessel within median nerve, 2 = two or three single or two confluent vessels and 3 = more than three single or more than two confluent vessels.

### Response to management

In addition to baseline assessment, every patient was assessed 1-week, 1-month, and 6-months after treatment. At every visit, every patient completed a questionnaire to record the change of their symptoms in response to treatment using a 0–10 VAS scale (El Miedany et al. 2006a) as well as a functional status assessment questionnaire (El Miedany et al. 2006b). US measures of CSA and flattening ratio as well as PD enhanced vascularity were recorded at 1-week, 1-month and 6-months visits. Nerve conduction studies were repeated at 6-months after treatment. All the patients who had neurological deficits (motor weakness, muscle wasting or sensory loss) were offered open surgical decompression of the carpal tunnel. The remaining patients were given the choice of having a conservative management approach including local steroid injection, splinting and exercise programs or surgical decompression of the carpal tunnel.

### Reproducibility of ultrasound findings

Inter-observer variability of B-mode and power Doppler findings was determined by intraclass correlation coefficient (ICC) and linear weighted κ coefficient, respectively, based on data from 34 patients’ wrists analysed by two ultrasonographers at one visit. Intra-observer variability was investigated by ICC using data from 30 patients.

### Control group

112 healthy subjects of matching sex and age were assessed as a control group. In addition to neurological examination, every subject had nerve conduction studies as well as Gray-scale and PD ultrasonography of their wrists and median nerve.

### Statistical analysis

Data collected were revised and introduced to a PC for statistical analysis. Quantitative data are presented as means and standard deviation while categorical data as frequency and percentage distribution. Sample size and distribution permitted the use of parametric tests as student-t test for independent 2 groups’ comparison in case of interval data or ANOVA if more than 2 groups are included. Post-hoc Tukey test is used to test individual groups differences. Pearson chi-square test was used to test association between 2 categorical variables, with Yates correction in case of 2 × 2 tables and Fisher Exact if less than 5 observations are encountered in one of the cells of the table. P value is always set at 0.05. Pearson correlation coefficient is used to test correlation between parametric quantitative variables while Spearman's is replaced if non parametric (scores). Multiple linear regression analysis was conducted to assess for independent biomarker(s) of prognosis (improvement at 6-months follow up). Different models were compared to achieve the best likelihood ratio. R2 was calculated as a measure of predictability of the model to the response. Scatterplot displays the correlation of the percent of improvement at 6 months and the unstandardized percent predicted by the model. All statistical manipulation and analyses were performed using the 15th version of SPSS.

## Abbreviations

CTS: Carpal tunnel syndrome
US: Ultrasound
PD: Power Doppler
NCS: Nerve conduction studies
MN: Median nerve
PROMs: Patient reported outcome measures
CSA: Cross sectional area

## References

[CR1] Abicalaf CA, de Barros N, Sernik RA, Pimentel BF, Braga-Baiak A, Braga L, Houvet P, Brasseur JL, Roger B, Cerri GG (2007). Ultrasound evaluation of patients with carpal tunnel syndrome before and after endoscopic release of the transverse carpal ligament. Clin Radiol.

[CR2] Al-Qattan MM, Bowen V, Manktelow RT (1994). Factors associated with poor outcome following primary carpal tunnel release in nondiabetic patients. J Hand Surg Br.

[CR3] Aroori S, Spence RA (2008). Carpal tunnel syndrome. Ulster Med J.

[CR4] Bessette L, Keller RB, Fossel AH, Mooney N, Katz JN (1997). Patients’ preferences and satisfaction following carpal tunnel release. J Hand Surg Am.

[CR5] Bland JD (2000). A neurophysiological grading scale for carpal tunnel syndrome. Muscle Nerve.

[CR6] Colak A, Kutlay M, Pekkafali Z, Saraçoglu M, Demircan N, Simşek H, Akin ON, Kibici K (2007). Use of sonography in carpal tunnel syndrome surgery. A prospective study. Neurol Med Chir (Tokyo).

[CR7] De Stefano F, Nordstrom DL, Vierkant RA (1997). Long-term symptom outcomes of CTS and its treatment. J Hand Surg Am.

[CR8] Eason SY, Belsole RJ, Greene TL (1985). Carpal tunnel release: analysis of suboptimal results. J Hand Surg Br.

[CR9] El Miedany Y, Aty S, Ashour S (2004). Ultrasonography versus nerve conduction study in patients with carpal tunnel syndrome: substantive or complementary tests?. Rheumatology (Oxford).

[CR10] El Miedany YM, Youssef SS, Mehanna AN, Meky F, Ashour S (2006). A new self-administered questionnaire for global assessment of symptoms severity and functional status of patients with carpal tunnel syndrome. Ann Rheum Dis.

[CR11] El Miedany Y, Ashour S, Youssef S, Mehanna A, El Gaafary M (2006). Validation of the modified self-administered questionnaire for assessment of functional status in patients with carpal tunnel syndrome. Arthritis Rheum.

[CR12] El Miedany Y, Ashour S, Youssef S, Mehanna A, El Gaafary M (2007). Sensitivity to change of the carpal tunnel syndrome global severity scoring index. A prospective study. Rheumatology (Oxford).

[CR13] El Miedany Y, Ashour S, Youssef S, Mehanna A, Meky F (2008). Clinical diagnosis of carpal tunnel syndrome: old tests–new concepts. Joint Bone Spine.

[CR14] El Miedany Y, El Gaafary M, Youssef S, Nasr A (2014). Gray scale and power Doppler ultrasound assessment of the median nerve: a biomarker that can help in setting a treat to target approach tailored for carpal tunnel syndrome patients. Ann Rheum Dis.

[CR15] Evans K, Roll S, Volz K, Freimer M (2012). Relationship Between Intraneural Vascular Flow Measured With Sonography and Carpal Tunnel Syndrome Diagnosis Based on Electrodiagnostic Testing. Ultrasound Med.

[CR16] Ferraccioli G, Alivernini S, Gremese E (2013). Biomarkers of Joint Damage in Rheumatoid Arthritis: Where Are We in 2013?. J Rheumatol.

[CR17] Hagberg M, Nystrom A, Zetterlund B (1991). Recovery from symptoms after carpal tunnel syndrome surgery in males in relation to vibration exposure. J Hand Surg Am.

[CR18] Hammer HB, Hovden IA, Haavardsholm EA, Kvien TK (2006). Ultrasonography shows increased cross-sectional area of the median nerve in patients with arthritis and carpal tunnel syndrome. Rheumatology (Oxford).

[CR19] Higgs PE, Edwards D, Martin DS, Weeks PM (1995). Carpal tunnel surgery outcomes in workers: effect of workers’ compensation status. J Hand Surg Am.

[CR20] Jeong J, Yoon J, Kim S, Park BK, Won SJ, Cho JM, Byun CW (2011). Usefulness of ultrasonography to predict response to injection therapy in carpal tunnel syndrome. Ann Rehabil Med.

[CR21] Jordan R, Carter T, Cummins C (2002). A systematic review of the utility of electrodiagnostic testing in carpal tunnel syndrome. Br J Gen Pract.

[CR22] Katz JN, Gelberman RH, Wright EA, Abrahamsson S, Lew RA (1994). A preliminary scoring system for assessing the outcome of carpal tunnel release. J Hand Surg Am.

[CR23] Katz J, Losina E, Amick B, Fossel AH, Bessette L, Keller RB (2001). Predictors of Outcomes of Carpal Tunnel Release. Arthritis Rheum.

[CR24] Klauser AS, Halpern EJ, De Zordo T, Feuchtner GM, Arora R, Gruber J, Martinoli C, Löscher WN (2009). Carpal tunnel syndrome assessment with US: value of additional cross-sectional area measurements of the median nerve in patients versus healthy volunteers. Radiology.

[CR25] Kuhlman KA, Hennessey WJ (1997). Sensitivity and specificity of carpal tunnel syndrome signs. Am J Phys Med Rehabil.

[CR26] Kulick MI, Gordillo G, Javidi T, Kilgore ES, Newmeyer WL (1986). Long-term analysis of patients having surgical treatment for carpal tunnel syndrome. J Hand Surg Am.

[CR27] Lee CH, Kim TK, Yoon ES, Dhong ES (2005). Postoperative morphologic analysis of carpal tunnel syndrome using high-resolution ultrasonography. Ann Plast Surg.

[CR28] Levine D, Simmons BP, Koris MJ, Daltroy LH, Hohl GG, Fossel AH, Katz JN (1993). Development and validation of symptom severity and functional status scales for carpal tunnel syndrome. J Bone Joint Surg Am.

[CR29] Lundborg G, Dahlin LB (1996). Anatomy, function, and pathophysiology of peripheral nerves and nerve compression. Hand Clin.

[CR30] Lundborg G, Myers R, Powell H (1983). Nerve compression injury and increased endoneural fluid pressure: A miniature compartment syndrome. J Neurol Neurosurg Psychiatry.

[CR31] Mackinnon SE, Dellon AL, Hudson AR, Hunter DA (1984). Chronic nerve compression — an experimental model in the rat. Ann Plast Surg.

[CR32] Mallouhi A, Pultzl A, Trieb T, Piza H, Bodner G (2006). Predictors of carpal tunnel syndrome: accuracy of gray-scale and color Doppler sonography. AJR Am J Roentgenol.

[CR33] Mohammadi A, Afshar A, Etemadi A, Masoudi S, Baghizadeh A (2010). Diagnostic value of cross-sectional area of median nerve in grading severity of carpal tunnel syndrome. Arch Iran Med.

[CR34] Mondelli M, Passero S, Giannini F (2001). Provocative tests in different stages of carpal tunnel syndrome. Clin Neurol Neurosurg.

[CR35] Mondelli M, Filippou G, Gallo A, Frediani B (2008). Diagnostic utility of ultrasonography versus nerve conduction studies in mild carpal tunnel syndrome. Arthritis Rheum.

[CR36] Mondelli M, Filippou G, Aretini A, Frediani B, Reale F (2008). Ultrasonography before and after surgery in carpal tunnel syndrome and relationship with clinical and electrophysiological findings. A new outcome predictor?. Scand J Rheumatol.

[CR37] Muhlau G, Both R, Kunath H (1984). Carpal tunnel syndrome—course and prognosis. J Neurol.

[CR38] Naranjo A, Ojeda S, Rúa-Figueroa I, Garcia-Duque O, Fernández-Palacios J, Carmona L (2010). Limited value of ultrasound assessment in patients with poor outcome after carpal tunnel release surgery. Scand J Rheumatol.

[CR39] Phalen GS (1966). The carpal-tunnel syndrome: seventeen years’ experience in diagnosis and treatment of six hundred fifty-four hands. J Bone Joint Surg Am.

[CR40] Rahmani M, Ghasemi Esfe AR, Vaziri-Bozorg SM, Mazloumi M, Khalilzadeh O, Kahnouji H (2011). The ultrasonographic correlates of carpal tunnel syndrome in patients with normal electrodiagnostic tests. Radiol Med.

[CR41] Roll SC, Case-Smith J, Evans KD (2011). Diagnostic accuracy of ultrasonography vs electromyography in carpal tunnel syndrome: a systematic review of literature. Ultrasound Med Biol.

[CR42] Salerno DF, Franzblau A, Werner RA, Chung KC, Schultz JS, Becker MP, Armstrong TJ (2000). Reliability of physical examination of the upper extremity among keyboard operators. Am J Ind Med.

[CR43] Smidt MH, Visser LH (2008). Carpal tunnel syndrome: clinical and sonographic follow-up after surgery. Muscle Nerve.

[CR44] Strickland JW, Gozani SN (2011). Accuracy of in-office nerve conduction studies for median neuropathy: a meta-analysis. J Hand Surg Am.

[CR45] Torp-Pedersen ST, Terslev L (2008). Settings and artefacts relevant in colour/power Doppler ultrasound in rheumatology. Ann Rheum Dis.

[CR46] Wang LY, Leong CP, Huang YC, Hung JW, Cheung SM, Pong YP (2008). Best diagnostic criterion in high-resolution ultrasonography for carpal tunnel syndrome. Chang Gung Med J.

[CR47] Yu G-Z, Firrell JC, Tsai T-M (1992). Preoperative factors and treatment outcome following carpal tunnel release. J Hand Surg Br.

[CR48] Ziswiler HR, Reichenbach S, Vogelin E, Bachmann LM, Villiger PM, Jüni P (2005). Diagnostic value of sonography in patients with suspected carpal tunnel syndrome: a prospective study. Arthritis Rheum.

